# Reduced Number of Lymphocytes by X-ray Irradiation: A Problem in a Combination Therapy Trial that Elicits the Abscopal Effect in Preclinical Studies Using Electron Beam Irradiation

**DOI:** 10.7759/cureus.4142

**Published:** 2019-02-26

**Authors:** Shiro Kanegasaki, Takashi Yamashita, Tomoko Tsuchiya

**Affiliations:** 1 Radiation Oncology, National Center for Global Health and Medicine, Tokyo, JPN; 2 Radiation Oncology, Japan Radioisotope Association, Tokyo, JPN

**Keywords:** chemokine, ccl3 derivative, alarmins, hsp70, hmgb1, t cells, nk cells, x-rays, reactive oxygen species, electron beams

## Abstract

In preclinical studies with model animals, intravenous administration of a derivative of chemokine CCL3, named eMIP, after local electron-beam irradiation, not only enhanced tumor growth inhibition at a target site but also induced tumor killing beyond the treated site (a phenomenon known as the abscopal effect). eMIP works with alarmins such as high mobility group box 1 (HMGB1) and heat shock protein 70 (HSP70) released from overexpressed tumor cells by irradiation. These alarmins at the irradiated tumor bed trap injected eMIP and, by forming complexes with eMIP, play a key role to recruit and activate tumor inhibitory natural killer (NK) cells and CD4^+^ and CD8^+^ T cells. Tumor type-specific secretion of gamma interferon from splenocytes was also demonstrated, which may also activate NK cells. During Phase 1 clinical studies using X-rays, however, no apparent abscopal effect was observed. Instead, we saw frequent reduction in numbers of lymphocytes in the peripheral blood of irradiated patients. The reduced number of lymphocytes recovered poorly once depleted, in contrast to neutrophils, and persisted for months after the treatment. This might have affected outcome after combination treatment of irradiation and eMIP. To enhance host defense mechanisms during and after photon-beam (X-ray) radiotherapy of a deep-seated tumor, it seems essential to keep lymphocytes undamaged by eliminating reactive oxygen species that are formed in the peripheral blood during irradiation.

## Introduction and background

A malignant tumor may grow by evading the body's immuno-surveillance, in which tumor specific lymphocytes play an important part. Tumor cells in the early stages of development have low immunogenicity and in the later stages they acquire an ability to evade the body’s host defense system. Recent identification of T cell inhibitory signals, including PD-L1 in tumor cells, and the presence of suppressive cells and factors indicate that a host defense system, by itself, has the potential to control tumor growth, at least in part, if there is no impediment [1]. Even if immunological tolerance could be breached, however, the numbers of lymphocytes recruited from the peripheral blood to the target site may not be enough for stand-alone immunotherapy focusing on unique cells such as killer T cells. Some treatment to eliminate the bulk of tumor cells may be required in addition to immunotherapy, like in the case of antibiotic treatment for bacterial infections [2]. Although chemotherapy is a systemic treatment and can act on multiple tumor sites in the body, tumor cells with slower dividing rates respond to chemotherapy much more modestly. Since leukocytes divide at a much faster rate than tumor cells, usual chemotherapy creates the serious problem of myelo- and immuno-suppression. It has been shown that high dose sequential chemotherapy consistently induces severe leukocyte depletion and that these populations do not recover [3].

In contrast to systemic chemotherapy, one notable advantage of local therapy for removing the bulk of the tumor, such as ionizing irradiation and heat treatments generated by radiofrequency ablation, is that the host defense system remains intact and can act on evading tumor cells. Among local tumor treatments, radiotherapy is most commonly employed. However, tumor cells usually re-emerge under hypoxic conditions and express hypoxia inducible factor-1, which allows the cells to adapt to low-oxygen conditions [4,5]. This makes them insensitive to the ionizing radiation that mediates its effects mostly through reactive oxygen species (ROS) generated by radiolysis of water and molecular oxygen [6]. Slowly dividing cells or cells with low metabolic activity are also less sensitive to irradiation. Furthermore, metastases are a common complication of the majority of solid tumors and radiotherapy itself is not designed to control out-of-field metastases.

Under such circumstances, inflammation induced by local irradiation, with the concomitant recruitment of leukocytes, plays an essential role in remission of tumors and patient outcome. In fact, radiation results in a number of changes in the tumor bed that may enhance the efficacy of immune responses in the body, including upregulation of MHC Class I molecules and influx of antigen presenting cells such as dendritic cells and macrophages. These cells process antigens from necrotic tumor cells and present antigenic peptides to effector T cells [7]. At the same time, various alarmins such as heat shock proteins (HSPs) and high mobility group box-1 protein (HMGB1) increase in the irradiated tumor bed. Among them, HSP70 is released from the plasma membrane of the necrotic tumor cells and works to activate natural killer (NK) cells and to present associated peptide to antigen presenting cells [8-10]. On the other hand, HMGB1 over expressed in tumor cells is also released [11,12] and enhances inflammation by activating dendritic cells and tumor-specific T cells [13,14]. Unfortunately, these events, after irradiation are not enough to eradicate the remaining metastatic tumor cells. It may be possible that if the host defense system can be activated by some means, the system should promote eradication of tumor cells that evade the treatment field and those at the metastatic sites.

## Review

Combination radiotherapy induces the abscopal effect in preclinical studies

We found striking systemic effects after intravenous administration of eMIP (code name: ECI301) in preclinical studies after electron-beam irradiation, where tumor growth distant to the irradiated site was also inhibited [15]. eMIP is a 69 amino acid derivative of human chemokine CCL3 carrying a single amino acid substitution of Asp27 to Ara and has improved pharmaceutical properties. Receptors for this chemokine are CCR1 and CCR5, both of which are expressed in NK cells and CD4^+^ and CD8^+^ T cells. In the initial experiments, tumors were transplanted subcutaneously in the right flank of mice and ionizing radiation (6 MeV-electron beam) was delivered to the tumor-bearing area. Administration of eMIP (2 μg/mouse once a day for 3-5 consecutive days starting from 1 day after irradiation) effectively prevents tumor growth, resulting in complete remission of about half of the mice treated. Tumor growth was not observed when the same tumor was transplanted again into the cured mice and tumor type-specific secretion of gamma interferon from splenocytes was demonstrated [16], suggesting that tumor specific T cells are associated with tumor killing. Gamma interferon generated by T cells may also activate another effector cell such as NK cells. The effects were not restricted to a specific tumor type, since growth inhibition was observed with various mouse tumors, including Lewis lung carcinoma, Meth A fibrosarcoma and Colon26 adenocarcinoma.

When tumor cells were inoculated in both flanks and only one tumor-bearing area was exposed to radiation, tumor growth at this non-irradiated site was inhibited, indicating that eMIP administration potentiated the abscopal effect. This effect, observed in both BALB/c and C57BL/6 mice, was tumor-type independent, suggesting that both specific and non-specific (inert) immunities are associated with induction of the abscopal effect. We found that both CD4^+^ and CD8^+^ T cells, at least, infiltrated not only the tumor bed at the irradiated site but also the non-irradiated site. This effect was not apparent after the administration of eMIP without irradiation, or after irradiation without eMIP administration. The immunodepletion of any of NK cells, CD4^+^ T cells or CD8^+^ T cells, diminished anti-tumor effects induced by eMIP, indicating that these lymphocyte subsets play essential roles in tumor regression at both treated and non-treated sites.

Mechanistic insights from the combination radiotherapy with eMIP

Predicting the presence of molecules that work in synergy with eMIP in the irradiated tumor bed, we examined if intratumor injection of tumor lysate could recapitulate the effect of radiation in the combination treatment and induces the abscopal effect [16]. Mice received eMIP (2 μg/mouse) intravenously once a day for five consecutive days starting from immediately after injection of tumor lysate to the center of the tumor mass at the right flank. We found that tumor growth was inhibited not only at the injected site but also at the nontreated site. Tumor lysate was not necessary to be syngeneic and those from Colon 26 cells and other tumor cell lysates (such as from MethA fibrosarcoma cells) were effective in Colon 26 bearing mice. We found alarmin HMGB1 in the sonicated tumor lysate from Colon 26 cells both in the supernatant and the precipitate after centrifugation at 20,000 x g for 15 minute. We then examined if HMGB1 could replace tumor lysate. Tumor growth in both flanks was markedly inhibited by HMGB1 (instead of tumor lysate), injected to the right-side tumor, followed by intravenous injection of eMIP. The role of HMGB1 in combination treatment of irradiation and eMIP administration was further confirmed by intraperitoneal injection of anti-HMGB1 antibody (two times: three hours before irradiation along with the fist eMIP administration). The neutralizing antibody diminished the cooperative effects of irradiation and eMIP, and tumor growth at the non-irradiated left flank as well as at the irradiated right flank became comparable with that without treatment (control).

**Figure 1:**
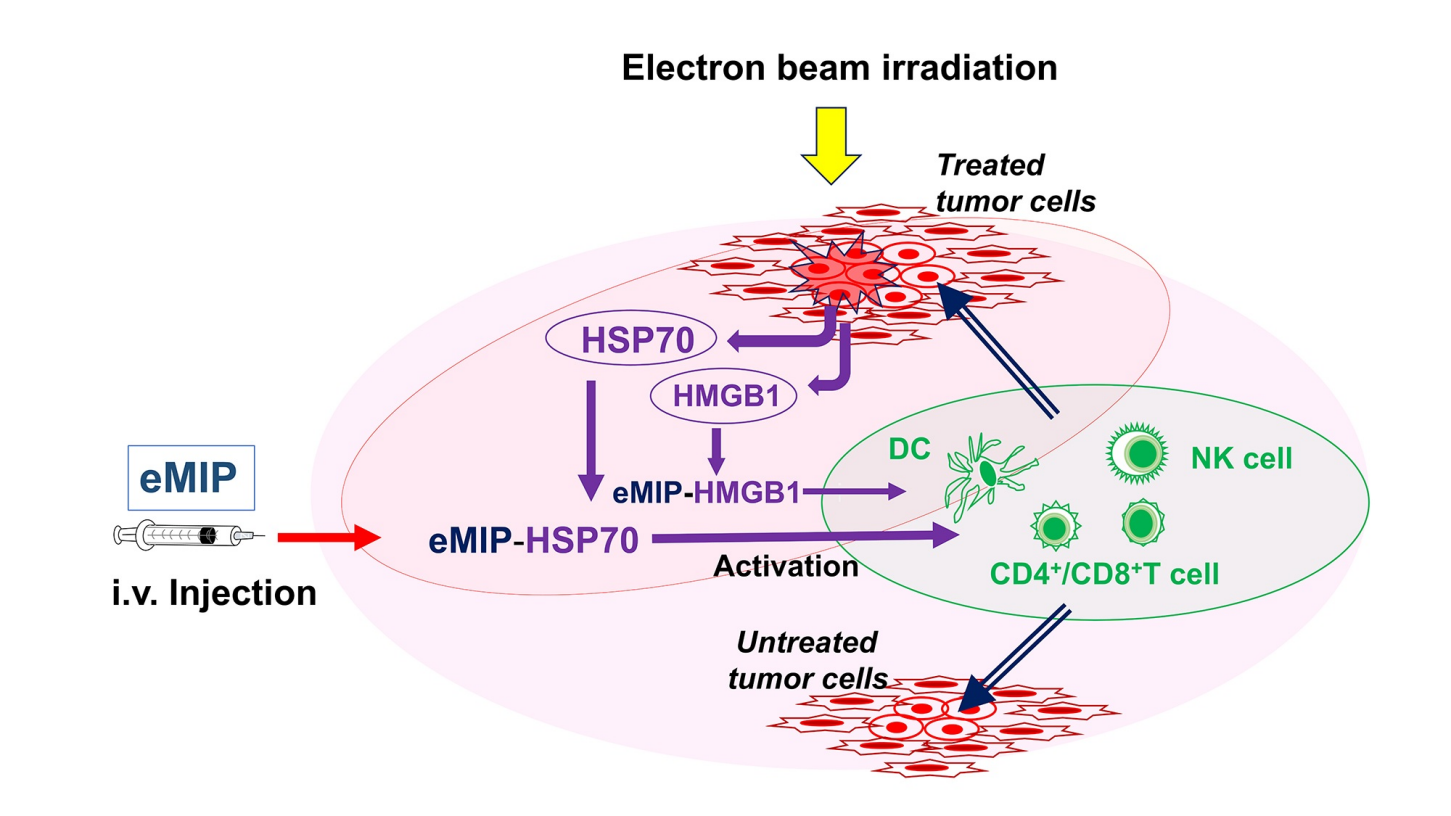
Action mechanism of the combination therapy. Ionizing irradiation (electron beam) induces apoptosis and necrosis of dividing tumor cells. Heat shock protein 70 (HSP70) and high mobility group box-1 protein (HMGB1), over expressed in these cells, are released, which trap intravenously injected circulating eMIP. eMIP/HSP70 and eMIP/HMGB1 complexes activate immune cells such as natural killer (NK) cells, CD4^+^ and CD8^+^ T cells, directly or indirectly, which eradicate tumor cells that may evade the treatments and those distal to the target sites. Dendritic cells (DC) may also play a role to activate NK cells and T cells. The same mechanisms could be elicited with other forms of ionizing radiation such as photons (X-rays), since no difference has been demonstrated in the biological effectiveness of the different forms of ionizing radiation.

It has been reported that HSPs are released from dying and necrotic tumor cells by irradiation followed by the late release of HMGB1 [17-19]. We next tested if HSP70 could also recapitulate the effect of radiation in the combination treatment with eMIP and induce the abscopal effect. Like in the case of HMGB1, results of similar experiments showed that tumor growth in both flanks was markedly inhibited by the combination treatment of HSP70 injected to the right-side tumor and intravenously administered eMIP. This effect was not observed with HSP60 or HSP90 instead of HSP70. Furthermore, mice, whose Toll-like receptor 4 (TLR4) function is defective, did not show antitumor responses to irradiation with eMIP administration. TLR4 is known to stimulate dendritic cells and other immune cells when reacted with HMGB1 or HSP70. As summarized in Figure 1, our results show that HSP70 and HMGB1 over-expressed by tumor cells are released into tumor beds upon irradiation, and trap intravenously-administered eMIP. The HSP70/eMIP and HMGB1/eMIP complexes enhance anti-tumor immunity. We have demonstrated in vitro that eMIP can bind both HSP70 and HMGB1. This may be why intravenously injected eMIP can work at tumor beds and remain active in immune cell activation. Unlike its bound form, free eMIP is cleared rapidly from the peripheral blood with a half-life of 1-1.5 hours due its small molecular mass (8 kDa) and fragmentation by the insulin-degrading enzyme with a comparable cleavage rate to insulin [20].

Phase 1 clinical trials

In accordance with the findings described above, Phase 1 clinical trials were started in the US (2009 and 2011, NCT01441115) and in Japan (2013) after Investigational New Drug (IND) approval by the United States Food and Drug Administration (US FDA) (IND 100,263, 2009 and IND 112,789, 20011). In these trials, the safety and tolerability of eMIP delivered in combination with external beam radiation to patients with metastatic cancer was assessed. Patients were treated with palliative radiotherapy in a standard manner, along with eMIP given daily during radiation. Ionizing radiation (X-rays) was delivered at a dose of 3 Gy per daily fraction, Monday through Friday, to a total dose of 30 Gy over two weeks. eMIP was delivered daily at the second week after each radiation treatment as an intravenous infusion. This dose of radiation provides palliation in most patients but is not considered curative and patients have a poor prognosis. This setting, however, offered a unique opportunity to test an agent that may enhance local therapy or enable the abscopal effect due to immune activation [21-23]. In our preclinical studies described above, ionizing radiation (6 MeV-electron beam) was delivered once before administration of eMIP. To confirm that fractionated radiation is also effective in inducing the abscopal effect, we performed a model experiment. Studies in BALB/c mice found that delivery of eMIP (administered five consecutive days starting from 1 day after initial irradiation) during a portion of a fractionated treatment schedule (2 Gy for five consecutive days) resulted in a similar efficacy as that seen in the unfractionated model (6 Gy). Given that the body weight of mice is 30 g, the dosage between 2 μg is approximately 70 μg/kg. Therefore, it was predicted that the optimal dosage for obtaining the anti-cancer effect would be lower than 100 μg/kg.

Effect of X-rays on peripheral lymphocytes

**Figure 2:**
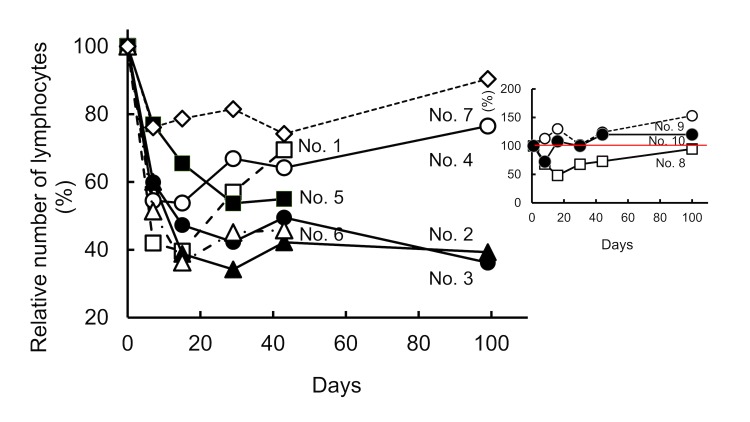
Relative number of lymphocytes before and after radiation treatment. Peripheral blood was obtained at day 0, 7, 16, 30, 40 and 99 and the number of lymphocytes was determined. Ionizing radiation (X-rays) was delivered at a dose of 3 Gy per daily fraction, at days 0-4 and 7-11. eMIP was administered at days 7-11 after each radiation treatment. Group 1: The patients in this group, maintained lymphocyte levels around or above 1000 cells/μl before irradiation. No. 1. Ovarian cancer (endometroid adenocarcinoma) with lymph node metastasis. Radiation: delivered to the supraclavicular and the pelvic lymph nodes No. 2. Colon cancer (moderately differentiated tubular adenocarcinoma) with lung lymph node metastasis. Radiation: delivered to the pelvic and the superficial cervical lymph nodes. No. 3. Breast cancer (moderately differentiated tubular adenocarcinoma) with liver metastasis. Radiation: delivered to the liver left lobe S4. No. 4. Submandibular gland cancer (basal cell adenocarcinoma) with lung metastasis. Radiation: delivered to the left lung. No. 5. Colon cancer (high-differentiated adenocarcinoma) with liver metastasis. Radiation: delivered to the liver S4. No. 6. Sigmoid colon cancer with lymph node metastasis. Radiation: delivered to the supraclavicular and the pelvic lymph nodes. No. 7. Colon cancer (high differentiated adenocarcinoma) with lung metastasis. Radiation: derlivered to the right middle, the left lower and the upper lobes. Group 2 (Inset): The patients in this group had lower numbers of lymphocytes even before irradiation (590-730 cells/μl). No. 8. Ovarian cancer (granulosa cell tumor) mesenteric recurrence. Radiation: delivered to the left iliopsoas muscle and the pelvis. No. 9. Oropharynx cancer (squamous cell carcinoma) with lung and cervical lymph node metastasis. Radiation: delivered to the left upper lobe. No. 10. Ovarian cancer dissemination (serous papillary cystic carcinoma). Radiation: delivered to the left pelvis and the rectal and the abdominal walls.

A total of 10 patients with severe metastatic ovarian, colon, breast, submandibular gland, and oropharynx cancers received local fractionated radiation followed by intravenous administration of eMIP. Neither notable clinical side effects nor apparent abscopal effect was found up to the 100 μg/kg dose level (Kashiwara et al., manuscript in preparation). We found, however, that local X-ray irradiation frequently reduces the number of lymphocytes in the peripheral blood (Figure 2), which possibly affects outcome after radiation treatment. The treated patients (brief information in Figure 2 legend) can be separated into two groups: those in the first group (seven out of 10) maintained lymphocyte levels around or above 1000 cells/μl before irradiation, which became reduced by 34-74% immediately after radiation treatment using the protocol described above (Figure 2). The reduced number of lymphocytes in existing (surviving) patients had not recovered by three months after irradiation except one case (Recovered by 94%). The patients in the second group (three out of 10) had lower numbers of lymphocytes even before radiation treatment (maximum 730 cells/μl), but the numbers were not reduced or, even when reduced, recovered to the original (low) level by three months after treatment (Figure 2). The results suggest that there are roughly two populations of lymphocytes in the peripheral blood with different sensitivity to irradiation. All patients had received chemotherapy and/or other therapies (immunotherapy, hormonotherapy and/or radiotherapy) before the trial.

More typically, lymphocyte reduction was seen in the peripheral blood in another colon cancer patient who had received surgical resections but not received chemotherapy before radiation treatment. In this case, using an intensity modulated radiation therapy (IMRT) technique, radiation was delivered to the paraaortic lymph node at a dose of 2 Gy per daily fraction, Monday through Friday, to a total dose of 56 Gy; and eight weeks after IMRT, using a stereotactic body radiotherapy (SBRT) technique, delivered to the left lower lobe nodule at a dose of 12 Gy per daily fraction to a total dose of 48 Gy. Before radiation treatment, the average numbers of lymphocytes and total leukocytes, respectively, in the peripheral blood shown as “mean (SD)” were 1974 (163) and 6112 (966) cells/μl (n = 5). After the patients received 38 Gy irradiation, the numbers of lymphocytes and total leukocytes were reduced to 987 (55) and 4310 (173) cells/μl (n = 4), respectively. The lymphocyte counts failed to recover even 3.5 months after the radiation treatment: 735 (7) cells/μl (n = 2), by which time the neutrophil/lymphocyte ratio had increased to 4.9 from 1.8 before the treatment.

Discussion

In mouse models, as described above, the abscopal effect was successfully induced by intravenously-administered eMIP after electron-beam irradiation on subcutaneously growing tumor cells. Nevertheless, no apparent abscopal effect was observed in 10 patients who received X-ray irradiation on various deep solid tumors followed by eMIP administration up to the 100 μg/kg dose level. In a similar combination radiotherapy using granulocyte-macrophage colony-stimulating factor (GM-CSF) reported in 2015 [24], abscopal responses were produced in 11 patients out of 41 enrolled patients. GM-CSF is a potent stimulus of dendritic cell maturation, although neutralizing autoantibodies against this cytokine are known to have caused autoimmune disease known as idiopathic pulmonary alveolar proteinosis [25]. The major differences between the clinical studies besides the immune stimuli employed (eMIP/GM-CSF) are the mode of administration of stimuli and radiation source. They injected the stimulus subcutaneously to the tumor bed (GM-CSF: 125 μg/m^2^ injected daily for two weeks) whereas we administered the stimulus intravenously (eMIP: up to 100 μg/kg). However, as shown, eMIP can be trapped by HSP70 and HMGB1 in the tumor bed and exert their effects there. Therefore, the difference in mode of administration was offset. Regarding the radiation source, we originally thought that the relative biological effect of electrons is comparable to that of photons (X-rays), and planned Phase 1 clinical studies were designed to use X-rays for irradiation on deep-seated tumors. In contrast, the researchers of the GM-CSF group used electron-beam irradiation on superficial metastatic lesions (35 Gy in 10 fractions, over two weeks), as in our preclinical studies in which the abscopal effect was induced efficiently.

We next observed reduced numbers of peripheral lymphocytes in patients from our clinical studies. In a total of seven out of 11 patients, whose lymphocytes in the peripheral blood were either more than 1000/μl but reduced significantly after irradiation, or lower than 700/μl when the treatment was started. Our present hypothesis is that the lymphocytes sensitive to chemotherapy and/or radiotherapy (reduced portion) play essential roles in the abscopal effect. It was reported by meta-analysis of 100 studies comprising 40,559 patients that a neutrophil/lymphocyte ratio greater than 4 could be a marker for poor prognosis [26]. Since lymphocytes are highly sensitive to ionizing radiation and, in contrast to neutrophils, poorly recover once depleted, the reduced number of lymphocytes seems to be responsible for raised neutrophil/lymphocyte ratios and poor patient prognosis in radiotherapies. We showed in our animal studies that various antitumor chemotherapeutic agents (such as docetaxel), which induce lymphopenia [27,28], diminished the effect of the combination treatments of irradiation and eMIP [15]. The success of combination treatments thus depends on the integrity of the host defense mechanism.

**Figure 3:**
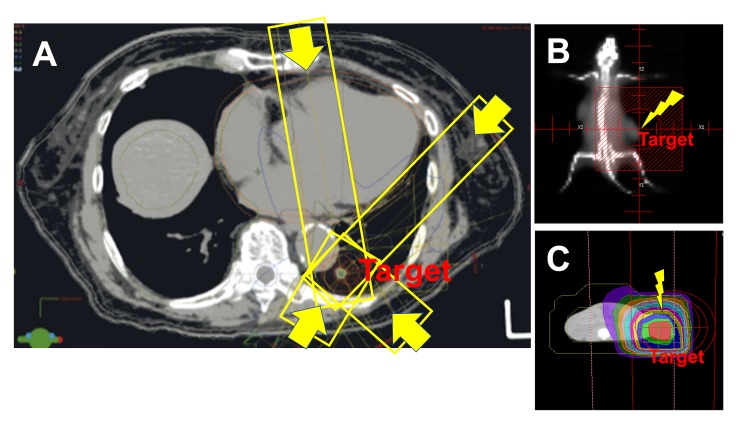
Difference between photon and electron beam irradiations in radiotherapies. (A) Computed tomography (CT) of a lung cancer patient receiving stereotactic body radiotherapy (SBRT). Four representative directions of X-rays (out of 12) are marked with yellow boxes and arrows. When the photon beam (X-ray) is traversing the lung, it encounters alveoli, each of which is surrounded by a capillary network so extensive that it forms an almost continuous sheet of surrounding blood. Therefore, regardless whether lung or other tissues, harmful reactive oxygen species (ROS) seem to be produced in the peripheral blood, which are considered to reduce lymphocyte number directly or indirectly. (B) Field of radiation and (C) CT simulation of the irradiated field in a mouse model used in our preclinical studies. Tumor cells were implanted subcutaneously in both flanks. Electron beams were derivers to the right flank from just above the tumor. The beams are only effective for subcutaneously growing tumor and scarcely affect other tissues. In contrast to X-rays, electron beams are known to have a finite range, after which dose falls off rapidly, sparing underling healthy tissue with extensive blood vessel networks.

In conventional photon beam radiation therapy (X-rays), the radiation dose is delivered at the surface of the body and passes through the body to reach the tumor. Various modes of X-ray irradiation using linear accelerators such as IMRT, SBRT and image guided-radiotherapy (IGRT) have been contrived to deliver precise radiation doses to a tumor to minimize the effects on surrounding or adjacent normal tissues. In these treatments, combinations of multiple intensity-modulated fields emit from different beam directions (Figure 3A). However, these therapies cannot exclude the dose of radiation that, on its way to the target tumor, encounters normal tissues at higher radiation levels. Compared with mouse models (Figure 3B), in which electron beams are delivered by a linear accelerator just above the subcutaneously implanted tumor in the flank [15], clinically delivered X-rays must pass through networks of blood vessels to reach the deep target site where tumors form. Since the dose decreases with increasing thickness of tissues, blood vessel networks in front of the target tumor are exposed to higher doses of ionizing radiation. It is known that ionizing radiation mediates its effects mostly through ROS such as hydroxyl radicals, which are generated by water and oxygen radiolysis.

Until now scant attention has been paid to the irradiation effect during treatment on the circulating blood. The flow rate of circulating blood in capillaries is slow (0.3 mm/sec) [29] while molecular oxygen is rich. Therefore, harmful ROS and their reaction products, including lipid peroxides, are formed in the blood vessels during radiation treatment and transferred to bone marrow and lymphatic organs. ROS and their products destroy lymphocytes and inhibit lymphopoiesis and development. This, and the presence of a lymphocyte fraction highly sensitive to ionizing radiation found by our clinical studies, are likely to be reasons why X-ray radiotherapy caused frequent reduction of peripheral lymphocytes and the failure in abscopal effect induction in our clinical studies using X-rays. During preparation of this article, we came across two papers very recently published [30,31], showing that radiation-induced lymphopenia is the result of direct toxicity to circulating lymphocytes as they traverse the irradiated field, which support our present findings and hypothesis.

It has been reported that supplemental antioxidants do not counteract radiotherapy treatment for a wide variety of cancers and may significantly mitigate the adverse effects of that treatment [32]. If we could eliminate ROS formed in the peripheral blood during irradiation, or even prevent their formation, lymphocytes would remain undamaged and patient outcome should improve, since irradiation-induced inflammation with concomitant recruitment of leukocytes (especially lymphocytes) are linked to patient outcome. Such treatments should improve patient outcome, and under such conditions, combination treatments that enhance host defense mechanism would work most efficiently. To reduce ROS and lipid peroxidation in the peripheral blood during irradiation, intravenous administration of anti-oxidants may be effective. Such treatment should be evaluated focusing on prevention of lymphopenia. Candidate molecules include: SOD, catalase, glutathione peroxidase/glutathione, ascorbic acid, vitamin-E and hydrogen-infused water. Another possibility is hydrogen gas inhalation during irradiation, which is known to reduce ischemia-reperfusion injury [33,34]. It is known that accelerated generation of ROS is a potential mediator of reperfusion injury.

## Conclusions

Development of a treatment to avoid abrogating a potential abscopal effect in circulating lymphocytes is warranted to induce the abscopal effect constantly by the combination treatments in photon beam (X-ray) radiotherapy.
